# Brain structural and functional changes in childhood and adolescent obesity: a systematic review of MRI studies

**DOI:** 10.3389/fnhum.2026.1920260

**Published:** 2026-08-27

**Authors:** Hui Xu, Junshen Zhang, Yang Wang, Guanjinghui Xu, Junjie Zhao, Xiao Fu, Guanghui Bai, Bujian Pan

**Affiliations:** 1 Department of General Surgery, The Second Affiliated Hospital and Yuying Children's Hospital, Wenzhou Medical University, Wenzhou, China; 2 School of Mental Health, Wenzhou Medical University, Wenzhou, China; 3 Department of Radiology, The Second Affiliated Hospital and Yuying Children’s Hospital, Wenzhou Medical University, Wenzhou, China

**Keywords:** adolescents, children, brain structure, function, executive function, obesity

## Abstract

**Background:**

Adolescent obesity has emerged as a global public health crisis, with accumulating evidence suggesting that it involves not only peripheral metabolic dysfunction but also significant central nervous system remodeling. Given that childhood and adolescence represent a critical window for neurodevelopment, understanding these alterations is vital for effective intervention. This review aimed to systematically synthesize multimodal magnetic resonance imaging (MRI) findings regarding brain structural and functional changes in children and adolescents with obesity, and its underlying neurobiological mechanisms.

**Methods:**

Following PRISMA guidelines, there were in total 41 high-quality studies encompassing functional MRI, structural MRI, and diffusion tensor imaging modalities involved in this review.

**Results:**

Synthesis of the literature reveals a consistent “reward-control” imbalance. Functionally, adolescents with obesity exhibited hyper-responsivity in reward-related regions (e.g., striatum, insula) to food cues, coupled with diminished recruitment of executive control areas (e.g., prefrontal cortex). Structurally, obesity was associated with gray matter atrophy in the prefrontal and cingulate cortices, and lower white matter integrity in tracts connecting these regions (e.g., corpus callosum, uncinate fasciculus). These changes may be associated with a synergy of chronic neuroinflammation, insulin resistance, and dopamine pathway dysregulation, although the cross-sectional nature of most evidence precludes definitive causal inferences.

**Conclusion:**

Obesity-related brain remodeling constitutes a pathological basis that reinforces maladaptive eating behaviors. Future research should prioritize longitudinal designs and targeted neuro-interventions to mitigate long-term cognitive and metabolic consequences in children and adolescent with obesity.

## 1 Introduction

Obesity has emerged as a global public health crisis. According to the 2023–2024 WHO report, approximately 42% of adults (≈2.2 billion) are overweight or have obesity, with projections estimating a rise to 54% (≈3.3 billion) by 2035. Obesity is a core risk factor for numerous chronic conditions, including type 2 diabetes, metabolic syndrome, cardiovascular diseases, and certain malignancies (Kumar and Kelly, 2017). Notably, there is a significant trend toward younger onset ages. Over the past three decades, the prevalence of obesity among children and adolescents has surged from approximately 8% to over 20%, with nearly one-third of children aged 5–14 classified as overweight or having obesity (Jebeile et al., 2022). Beyond metabolic implications, obesity may adversely affect brain development and cognitive function (Sadler et al., 2023). The trend toward earlier onset implies that obesity may exert long-term influences during critical periods of neurodevelopment. Consequently, brain alterations in children and adolescents with obesity have become a central focus of current research (Kennedy et al., 2016).

The neurobiological mechanisms of obesity involve dysregulation in central energy balance, particularly within the hypothalamic arcuate nucleus, which integrates peripheral hormonal signals (leptin, insulin, ghrelin) and neural signals to regulate food intake and energy expenditure. Dysregulation of these signals may contribute to sustained appetite elevation and decreased energy expenditure. Additionally, imbalances in the reward system play a critical role; the dopamine reward pathway, involving the nucleus accumbens (NAc) and ventral tegmental area, is intensely activated by high-calorie food intake. Individuals with obesity often exhibit reduced dopamine D2 receptor availability (Johnson and Kenny, 2010), which may result in an elevated reward threshold and increased feeding impulse. Furthermore, obesity triggers hypothalamic microglial activation and the release of inflammatory factors (IL-1β, TNF-*α*), which may further impair hypothalamic neurons (Thaler et al., 2012).

Magnetic resonance imaging (MRI) is increasingly utilized to explore brain structural and functional changes in adolescents with obesity (Ou et al., 2015). The multi-modal nature of MRI can link structural with functional changes and behavioral performance, thereby constructing a complete explanatory chain from biological basis to behavior (Yau et al., 2012). A previous study found that, adolescents with obesity exhibit lower functional connectivity between the reward system (e.g., nucleus accumbens) and the cognitive control network (prefrontal cortex) (Pujol et al., 2021). Another study found that when exposed to high-calorie foods, adolescents with obesity showed enhanced activation in the executive function network (dorsolateral prefrontal cortex) but reduced activation in the insula (associated with interoception/satiety), suggesting reward sensitization and impaired inhibitory control (Carnell et al., 2017). Furthermore, significantly lower functional connectivity (FC) between the amygdala and prefrontal cortex weakens the neural support for emotional regulation. In food-induced decision-making tasks, adolescents with obesity demonstrate attenuated prefrontal inhibitory signals accompanied by striatal overactivation, pointing to impulsive eating behaviors that exacerbate obesity (Batterink et al., 2010). Structurally, several studies report that gray matter volume of the dorsolateral prefrontal cortex, middle frontal gyrus, and superior frontal gyrus are significantly lower in adolescents with obesity than in normal-weight peers (Islam et al., 2018). These regions are hubs for executive function and decision-making. Further research indicates that anterior cingulate cortex (ACC) volume is significantly reduced in obesity and negatively correlated with BMI, with faster weight gain associated with more pronounced gray matter atrophy (Kennedy et al., 2016). This is critical as the ACC is integral to conflict monitoring and emotional regulation. Mild volume reductions have also been observed in the thalamus and inferior parietal lobule. Additionally, previous research about diffusion tensor imaging (DTI) found that individuals with obesity show significantly lower fractional anisotropy (FA) in the corpus callosum, cingulum, and medial prefrontal tracts, suggesting compromised axonal integrity and myelination (Ou et al., 2015). Collectively, these changes compromise cognitive, executive, and emotional functions, further reinforcing unhealthy eating behaviors (Lowe et al., 2019).

However, existing literature is predominantly cross-sectional, which hinders the determination of causality between brain changes and obesity. Moreover, inconsistent control of confounding factors, the use of crude obesity metrics (mostly BMI rather than body fat or visceral fat), and limited sample sizes constrain the precision of findings. Given the diversity in study designs, there is currently no comprehensive review synthesizing MRI findings of brain changes specifically in pediatric populations, as most previous reviews have focused on adults. Therefore, this systematic review aimed to synthesize results across task-based functional MRI (fMRI), resting-state fMRI, structural MRI in children and adolescents with obesity, and to evaluate the strength and consistency of the evidence for obesity-related brain changes.

## 2 Methods

### 2.1 Search strategy

In this review, PubMed served as the primary database. The search strategy integrated medical subject headings and free-text terms related to “Obesity,” “Children/Adolescents,” and “magnetic resonance imaging/MRI” (see Table 1). To supplement electronic searches, reference lists of included studies were manually screened to identify relevant articles not captured initially.

**Table 1:** Search strategy for identifying articles on MRI studies in obesity.

Search strategy
1. Obesity
2. Overweight
3. Adolescents/Children
4. Excess BMI
5. Functional connectivity
6. Brain function/structure
7. Gray/White matter
8. Functional magnetic resonance imaging
9. Structural magnetic resonance imaging
10. Obesity search term:1OR 2OR 3OR 4
11. Neuroimaging search terms: 5OR OR6 OR7 OR8 OR 9
12. Final search terms: 10 AND 11

MRI, magnetic resonance imaging; BMI, body mass index.

### 2.2 Inclusion and exclusion criteria

This study aimed to systematically assess brain structural and functional changes related to obesity in children and adolescents. Included literature had to meet the following criteria: First, participants must be children or adolescents, and the study design must include a direct comparison between an obesity group (or obesity with comorbidities group) and a normal-weight control group. Second, to maximize the depth of the review and ensure homogeneity of included studies, this review only included literature based on MRI technology, excluding other neuroimaging methods. Specific imaging modalities covered include: resting-state functional MRI (rs-fMRI), tasking-state functional MRI, structural MRI-Gray Matter, and structural MRI-White Matter. To ensure the relevance of included literature and the quality of evidence, strict exclusion criteria were established. The following types of literature were excluded: studies that did not use MRI as the neuroimaging assessment tool or failed to directly investigate the association between obesity and brain structure/function in children and adolescents. Non-human studies based on animal models or *in vitro* experiments. Non-original research papers or publications unable to provide complete data, including review articles, systematic reviews, case reports, editorials, commentaries, and conference abstracts.

In accordance with standard pediatric guidelines, obesity in children and adolescents was defined using age- and sex-specific BMI percentiles (≥95th percentile) or BMI z-scores as reported in the original studies. Studies that reported absolute BMI thresholds (e.g., BMI ≥ 28 kg/m^2^) were only included if they explicitly specified that their sample consisted of adolescents (≥12 years of age), where adult criteria may be applicable.

### 2.3 MRI outcome measures

MRI measures focused on in this review are divided into two categories. One category is structural MRI, which adopted high-resolution T1-weighted 3D MPRAGE sequences and utilizes tissue segmentation algorithms (such as SPM/CAT12) for voxel-based morphometry or surface-based morphometry analysis to obtain indicators such as gray matter volume, density, cortical thickness, and surface area. White matter integrity is obtained through DTI, calculating indicators such as FA and mean diffusivity, often analyzed using tract-based spatial statistics or fiber tracking techniques. Another category is functional MRI, which was based on the blood-oxygen-level-dependent effect. After standard preprocessing workflows (e.g., slice timing correction, head motion correction, normalization), resting-state fMRI is used to analyze brain network connectivity, while task-based fMRI is used to localize brain region activation under specific cognitive or behavioral stimuli.

### 2.4 Study selection and data extraction

**Figure 1:**
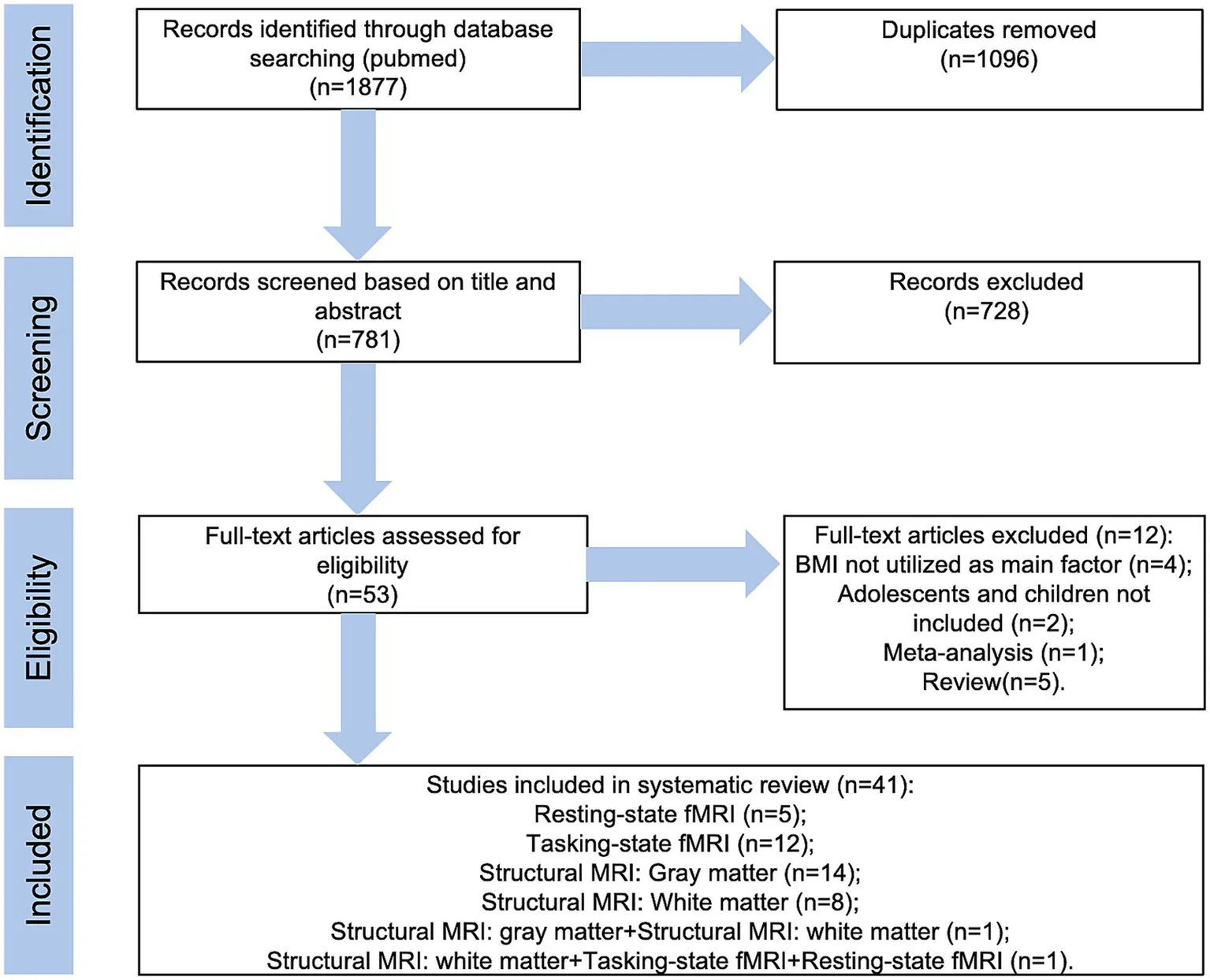
Flowchart of the literature search and selection process. This diagram details the study selection process, ranging from the initial PubMed database search, removal of duplicates, and screening to the final inclusion in the systematic review. MRI, magnetic resonance imaging; fMRI, functional magnetic resonance imaging.

Two reviewers independently performed literature screening and data extraction. First, titles and abstracts were screened based on the research topic to exclude studies that clearly did not meet the criteria. Subsequently, the full text of the literature retained from the initial screening was reviewed. Disagreements were resolved through discussion or consultation with a third party. Two reviewers independently recorded information from the included studies in separate forms, calibrating based on the review topic to maximize consistency. The following information was recorded for each study: publication details (publication name, author name, age, publication year, etc.); imaging modality (structural MRI-White matter, structural MRI-Gray matter, resting-state fMRI, task-based fMRI); satisfaction of inclusion/exclusion criteria; data analysis strategy used; sample size; and demographic characteristics of the sample (age, gender). From the initially retrieved 1,877 articles, 41 relevant studies meeting the inclusion criteria were finally selected (see PRISMA flow diagram for the screening process, Figure 1).

### 2.5 Quality assessment

Methodological quality was evaluated based on the study design’s capacity to elucidate the relationship between childhood or adolescent obesity and brain structure or function. In this context, “quality” does not denote the intrinsic merit of a study but rather its pertinence and methodological rigor in addressing this review’s specific research question.

The risk of bias was assessed using the risk of bias in non-randomized studies of exposures tool. This tool evaluates observational studies by benchmarking them against a theoretical “target randomized controlled trial” across seven domains: (a) Confounding: adequacy of control for key covariates (e.g., age, sex, intracranial volume). (b) Participant selection: representativeness of the sample relative to the target population. (c) Exposure classification: accuracy of the obesity measurement. (d) Deviations from intended exposures: impact of concurrent interventions affecting brain structure/function post-obesity onset. (e) Missing data: impact of participant attrition or incomplete follow-up. (f) Outcome measurement: blinding of MRI data analysis. (g) Selection of reported results: selective reporting of significant findings.

In terms of risk of bias, studies were classified as high risk (“+”) if at least one domain was rated as “high risk” or if multiple domains raised “some concerns” that substantially compromised result validity. All other studies were marked as low risk (“++”).

In terms of directness, this metric assessed the external validity and applicability of the study. A study was deemed to have low directness (“+”) if it failed significantly on the following criteria; otherwise, it was marked as high directness (“++”): (a) Demographic alignment (age, sex, ethnicity, nationality) with the review’s target population. (b) Consistency of obesity definitions (e.g., BMI z-score, percentiles) with this review. (c) Appropriateness of the normal-weight control group. (d) Relevance of neuroimaging outcomes to the review’s core interests. In terms of precision, this metric evaluated the statistical power and stability of the effect estimates. High precision (“++”) was awarded if the study met at least one high-quality standard: (a) a robust sample size (≥30 participants defined as high quality; 10–29 as moderate; <10 as low), or (b) the utilization of a longitudinal design. Otherwise, it was marked as low precision (“+”).

## 3 Results

### 3.1 Resting-state fMRI

In terms of resting-state fMRI, there were 6 studies involved. These studies mainly adopted cross-sectional designs, focusing on local brain activity indicators and FC patterns. Due to significant differences in data preprocessing and region of interest selection, results are summarized qualitatively.

Existing evidence indicates that childhood and adolescent obesity is closely related to significant reorganization of the brain’s intrinsic functional architecture. Multiple rs-fMRI studies revealed extensive functional abnormalities in reward networks, cognitive control networks, and somatosensory networks in individuals with overweight or obesity. Specifically, adolescents with overweight status showed weakened FC between key nodes within the salience network and default mode network (DMN), mainly involving the ACC and middle temporal lobe-posterior cingulate/precuneus pathways. Meanwhile, FC between the middle temporal lobe and orbitofrontal cortex (OFC) was enhanced (Moreno-Lopez et al., 2016). This deviation suggests abnormal integration between interoceptive signal processing and reward evaluation networks. Within the reward circuit, adolescents with overweight showed enhanced network connectivity among the left OFC, ventral striatum, and anterior insula; the connectivity strength between the NAc and the midbrain was positively correlated with emotional eating behavior, revealing pathological reward-driven mechanisms (Martín-Pérez et al., 2019). Conversely, FC in cerebellar and prefrontal regions appeared weakened, suggesting impaired synergy between motor control and cognitive regulation.

Children with obesity showed significantly elevated fractional amplitude of low-frequency fluctuations in the left insula, left superior temporal gyrus, left middle frontal gyrus, and right middle cingulate gyrus (Liu et al., 2025). Correlation analysis showed these abnormal activity levels were closely related to visceral fat area and liver function indicators, suggesting peripheral metabolic disorders may directly regulate the baseline spontaneous activity of central neurons. Additionally, a comorbidity study found that normal-weight adolescents with bipolar disorder had higher cerebral blood flow in reward regions than the obesity bipolar group and healthy controls, indicating weight status may modulate neurovascular coupling in psychiatric populations (Grigorian et al., 2022).

Furthermore, research found that the negative coupling between the DMN and the executive control network (ECN) was weakened, potentially leading to declined self-regulation abilities (Pujol et al., 2021). As BMI increased, cortico-subcortical FC in the reward system tended to weaken, while local connectivity between the somatosensory cortex and superior parietal lobule enhanced (Pujol et al., 2021). Graph theory analysis further showed global efficiency decreases in the DMN, attention network, and reward network in individuals with obesity, specifically accompanied by reduced transmission efficiency in cortico-thalamic pathways. These functional alterations were correlated with structural reductions in the anterior cingulate and prefrontal cortices in the same sample (Brooks et al., 2023), suggesting a potential structural-functional relationship. More details were provided in Table 2.

**Table 2:** Resting-state fMRI studied investigating brain functions in children or adolescents.

Study	Participants		Design	Main findings
	Subjects (*n*)	Age (years old)		
Moreno-Lopez et al. (2016)	Excess weight (60): 38F/22M; Normal weight (55): 32F/23M	12–17	Case–control study with a cross-sectional design	(1) The connectivity between the insula and the anterior cingulate cortex was reduced in comparison to controls, as was the connectivity between the middle temporal gyrus and the posterior cingulate cortex and cuneus/precuneus. (2) The middle temporal gyrus displayed increased connectivity with the orbitofrontal cortex in comparison to controls.
Grigorian et al. (2022)	Bipolar disorder (70): 25OB/55NW; HC (61)	13–20	A cross-sectional neuroimaging study employing arterial spin labeling to measure cerebral blood flow	Youth with comorbid bipolar disorder and overweight (BDOW/OB) and healthy controls (HC) showed lower cerebral blood flow (CBF) in key reward and subcortical regions compared to normal-weight bipolar youth (BDNW)
Liu et al. (2025)	Obesity (59); Normal weight (48)	6–16	Cross-sectional case–control study	(1) Children with obesity had higher fALFF values in the left insula, left superior temporal gyrus (STG), left middle frontal gyrus and right middle cingulate gyrus (MCG). (2) In the obesity group, fALFF values in the left STG positively correlated with visceral and subcutaneous adipose tissue area and verbal comprehension index. (3) fALFF values in the right MCG positively correlated with alanine aminotransferase and aspartate aminotransferase levels.
Martín-Pérez et al. (2019)	Excess weight (56)	10–19	Cross-sectional case–control study	(1) EW showed higher functional connectivity in the LH-orbitofrontal cortex, ventral striatum, anterior insula, and in the MH-middle temporal cortex networks. (2) EW also showed lower connectivity in the LH-cerebellum, and in the MH-middle prefrontal, pre-, and postcentral gyri networks. (3) In EW, higher connectivity of the LH-nucleus accumbens and LH-midbrain networks were associated with stress response. (4) Higher connectivity in the LH-midbrain was also associated with a greater presence of emotional eating behaviors in EW.
Pujol et al. (2021)	Children (218): 113F/105M	8–12	Cross-sectional observational study	(1) Higher body mass index was associated with weaker connectivity between the cortical and subcortical elements of the reward system. (2) Higher BMI percentiles were associated with stronger local functional connectivity in a region involving the somatosensory cortex at the cortical representation of the body and the superior parietal cortex.
Brooks et al. (2023)	Children (4921): 2572F/2349M	9–11	Cross-sectional magnetic resonance study	(1) Those with obesity or overweight had lower topological efficiency, resilience, connectivity, connectedness and clustering in Default-Mode, dorsal attention, salience, control, limbic, and reward networks. (2) Lower cortico-thalamic efficiency and connectivity were estimated only in youth with obesity. (3) Both groups had lower cortical thickness, volume and white matter intensity in these networks’ constituent structures, particularly anterior cingulate, entorhinal, prefrontal, and lateral occipital cortices.

fMRI, functional magnetic resonance imaging; F, female; M, male; BD, bipolar disorder; OB, obesity; NW, normal weight; HC, healthy controls; EW, excess weight; fALFF, fractional amplitude of low-frequency fluctuations; BMI, body mass index; LH, lateral hypothalamus; MH, medial hypothalamus.

### 3.2 Task-based fMRI

In terms of task-based fMRI, there were a total of 13 studies covering experimental paradigms food cue stimulation, inhibitory control, and decision-making. Due to task design heterogeneity, a unified effect size meta-analysis was not possible; results are described systematically by cognitive domain.

Task-based fMRI studies confirmed that adolescents with obesity exhibit significant neural activation pattern differences under specific cognitive and sensory tasks, characterized by a dual imbalance of “reward hyperactivation” and “control hypoactivation.” Cognitive deficits were observed in tasks involving fluid reasoning and visual–spatial working memory, correlated with structural and functional alterations in relevant circuits (Brooks et al., 2023). When facing food cues (e.g., taste cues or high-calorie food pictures), individuals with obesity showed overactivation of reward-related brain regions, primarily involving the insula, amygdala, and OFC (Bohon, 2017; Kerem et al., 2022; Moreno-Padilla et al., 2018; Rapuano et al., 2016). Some studies also noted hyperactivation in the dorsolateral prefrontal cortex in response to food pictures, linked to motivation and lower self-esteem (Davids et al., 2010). This high reactivity was evident not only during stimulus presentation but also as insufficient inhibition of reward regions in the post-prandial state (Bruce et al., 2010), suggesting satiety signals failed to effectively suppress impulse signals in the reward circuit—specifically, children with obesity showed elevated response to tastes in the insula and amygdala even when sated (Boutelle et al., 2015).

In contrast to the overactive reward system, adolescents with obesity showed significant activation reduction in the “prefrontal cortex (PFC),” a core region of the cognitive control network, during tasks involving food logo recognition and decision-making (Bruce et al., 2013). In risk decision tasks, individuals with obesity presented a pattern of decreased insula activation and enhanced midbrain response (Delgado-Rico et al., 2013; Verdejo-García et al., 2015); this neural dissociation may constitute the neural basis for impulsive decision-making and increased risk behaviors. Additionally, functional abnormalities in structures like the hypothalamus and hippocampus were observed, which may further affect energy homeostasis regulation and memory-driven eating behaviors (Mestre et al., 2017; Sewaybricker et al., 2019). In summary, brain functional changes in adolescents with obesity involve interactive dysregulation across reward, cognitive control, and decision-making systems, providing important imaging evidence for understanding their abnormal eating behaviors. More details were provided in Table 3.

**Table 3:** Task-based fMRI studied investigating brain functions in children or adolescents.

Study	Participants		Design	Main findings
	Subjects (*n*)	Age (years old)		
Brooks et al. (2023)	Children (4921): 2572F/2349M	8–11	Cross-sectional magnetic resonance study	Youth with obesity or overweight had lower scores in a task measuring fluid reasoning – a core aspect of cognitive function, which were partially correlated with topological changes.
Bohon (2017)	Excess weight (8): 5F/3M; Normal weight (10): 8F/2M	6–8	Cross-sectional case–control study	(1) Greater response to milkshake taste receipt in children with overweight in the right insula, operculum, precentral gyrus, and angular gyrus, and bilateral precuneus and posterior cingulate. (2) No group differences were found for brain response to a visual food cue.
Moreno-Padilla et al., 2018	Excess weight (38); Normal weight (39)	14–19	Cross-sectional observational study	Adolescents with excess weight showed higher brain activation in frontal, striatal, insular and mid-temporal regions during choices between appetizing and standard food cues.
Rapuano et al. (2016)	Excess weight (18); Normal weight (19)	12–16	Functional magnetic resonance imaging study	(1) Compared with non-food commercials, food commercials more strongly engaged regions involved in attention and saliency detection (occipital lobe, precuneus, superior temporal gyri, and right insula) and in processing rewards and left orbitofrontal cortex. (2) Activity in the left OFC and right insula further correlated with subjects’ percent body fat at the time of the scan. (3) Higher-adiposity adolescents mentally simulate eating behaviors and offers a potential neural mechanism for the formation and reinforcement of unhealthy eating habits.
Verdejo-García et al. (2015)	Excess weight (36); Normal weight (44)	12–18	A functional magnetic resonance imaging study incorporating behavioral measures and psychological scales	(1) Adolescents with excess weight compared to controls display significantly decreased activation of anterior insula, anterior cingulate, and midbrain during decisions about Unfair versus Fair offers. (2) Excess weight subjects show lower sensitivity to reward and more maturity fears, which correlate with insula activation.
Sewaybricker et al. (2019)	Obesity (11); Healthy weight (9)	9–17	Cross-sectional case–control study	(1) Children with obesity had longer T2 relaxation times, consistent with gliosis, in the mediobasal hypothalamus (MBH) compared to controls. (2) Longer T2 relaxation times correlated with measures of higher adiposity, including visceral fat percentage. (3) Mean glucose-induced hypothalamic blood oxygen-level dependent signal change did not differ between groups. (4) Mean left MBH T2 relaxation time negatively correlated with glucose-induced hypothalamic signal change.
Boutelle et al. (2015)	Obesity (10); Healthy weight (13)	8–12	Cross-sectional case–control study	(1) A ROI analysis revealed an elevated BOLD response to taste within the bilateral insula and amygdala in OB children relative to HW children. (2) Whole-brain analyses revealed a group by condition interaction within the paracingulate, medial frontal, middle frontal gyri and right amygdala: *post hoc* analyses suggested an increased response to sucrose for OB relative to HW children, whereas HW children responded more strongly to water relative to sucrose. (3) OB children, relative to HW, tended to recruit the right putamen as well as medial and lateral frontal and temporal regions bilaterally.
Bruce et al. (2010)	Obesity (10); Healthy weight (10)	11–16	Cross-sectional case–control study	(1) Both groups of children showed brain activation to food images in the limbic and paralimbic regions (PFC/OFC). (2) The group with obesity showed significantly greater activation to food pictures in the PFC (pre-meal) and OFC (post-meal) than the HW group. (3) The group with obesity showed less post-meal reduction of activation (vs pre-meal) in the PFC, limbic and the reward-processing regions, including the nucleus accumbens.
Davids et al. (2010)	Excess weight (22); Normal weight (22)	9–17	Cross-sectional case–control study	(1) Children with Obesity showed higher activation of the dorsolateral prefrontal cortex (DLPFC) in response to food pictures. (2) DLPFC activation was negatively correlated with self-esteem. (3) Normal-weight children showed higher activation of the caudate and hippocampus specific to food pictures, and of the anterior cingulate cortex and thalamus to visual cues in general. (4) In response to food stimuli, children with obesity showed a heart rate deceleration correlating positively with activation of the ventrolateral prefrontal cortex.
Bruce et al. (2013)	Obesity (10); Healthy weight (10)	10–14	Cross-sectional case–control study	Compared with the healthy weight children, children with obesity showed significantly less brain activation to food logos in the bilateral middle/inferior prefrontal cortex, an area involved in cognitive control.
Mestre et al. (2017)	Obesity (12); Healthy weight (13)	8–12	Cross-sectional case–control study	(1) Children with OB, relative to HW, showed reduced left hippocampal volume and greater response to taste in three clusters within the left hippocampus. (2) Activation within the hippocampus was associated with eating in the absence of hunger and two subscales on a measure of eating behaviors.
Delgado-Rico et al. (2013)	Obesity (21); Excess weight (15); Normal weight (16)	12–17	Cross-sectional case–control study	(1) Excess weight adolescents, compared to normal weight controls, showed decreased left insular and increased midbrain activations during anticipation of risky choices. (2) Excess weight adolescents showed increased activations of the inferior frontal gyrus, parahippocampus, thalamus, and posterior brain regions after reward receipt.
Kerem et al. (2022)	Obesity/Overweight (11); Health weight (12)	11–23	Cross-sectional observational study	Participants with ARFID and OV/OB demonstrated significant hyperactivation in response to HCF (vs. objects) in the orbitofrontal cortex (OFC) and anterior insula compared with HW participants with ARFID.

fMRI, functional magnetic resonance imaging; F, female; M, male; BOLD, blood oxygen level dependent; OFC, orbitofrontal cortex; OB, obesity; ROI, region of interest; HW, healthy weight; PFC, prefrontal cortex; DLPFC, dorsolateral prefrontal cortex; MBH, mediobasal hypothalamus; ARFID, avoidant/restrictive food intake disorder; OV, overweight; HCF, high-calorie foods.

### 3.3 Structural MRI-gray matter

In terms of structural MRI-gray matter, there were 17 studies, which comprised 10 cross-sectional observational studies, 5 cross-sectional case–control studies, 1 tracking study, and 1 prospective study.

Evidence primarily points to a negative correlation between obesity and cortical thickness. Multiple large-scale cross-sectional studies consistently noted that higher BMI is significantly associated with reduced cortical thickness in prefrontal regions, mainly affecting the OFC, anterior cingulate gyrus, and superior frontal gyrus (Ronan et al., 2020; Ross et al., 2015). For example, a study in a large sample of 2,668 children found that elevated BMI was associated with impaired executive function, a relationship partially mediated by thinning of the PFC (including the OFC) (Ronan et al., 2020). However, another population-based study observed an opposite positive association in a sample of over 3,000 children (Steegers et al., 2021). This discrepancy suggests the relationship may be complexly regulated by developmental stage specificity or potential confounders. Research on gray matter surface area is scarcer, with only isolated studies reporting significant reductions in gray matter structural areas in children with obesity.

Research reveals a more complex, region-specific bidirectional change pattern. On one hand, longitudinal evidence shows accelerated decline in gray matter volume in the PFC, thalamus, and precentral gyrus in children with obesity over a two-year follow-up (Jiang et al., 2023). Multiple studies also reported volume atrophy in the anterior cingulate gyrus, medial OFC (Zhang et al., 2023), and superior/middle frontal gyri (Yokum et al., 2012), and other regions such as the cerebellum and temporal gyrus (Ou et al., 2015) in adolescents with obesity or overweight. Similar reductions in frontal gray matter were observed in specific subgroups, such as children with autism spectrum disorder and obesity (Cheng et al., 2023). Furthermore, a recent study demonstrated a positive coupling between cerebral blood flow and gray matter volume in visual and insular regions, suggesting vascular factors may underlie these structural changes (Brady et al., 2025). On the other hand, multiple independent studies observed volume increases in subcortical nuclei in adolescents with obesity, including the globus pallidus (de Groot et al., 2017), hippocampus (Moreno-López et al., 2012), amygdala (Nouwen et al., 2017; Perlaki et al., 2018), and olfactory bulb (Aytaç Kaplan et al., 2025; Karaoglan and Colakoglu Er, 2020). This coexistence of “atrophy” in cortical control regions and “hyperplasia” in subcortical reward/emotion regions may reflect specific pathophysiological remodeling imposed by obesity on brain structure. However, evidence regarding subcortical structures is not entirely uniform; other studies have reported reduced volumes or signal abnormalities in the hippocampus and amygdala, particularly in adolescents with metabolic comorbidities like type 2 diabetes (Mestre et al., 2020; Nouwen et al., 2017; Tirsi et al., 2013). For more details see Table 4.

**Table 4:** Structural MRI-gray matter studied investigating brain functions in children or adolescents.

Study	Participants		Design	Main findings
	Subjects (*n*)	Age (years old)		
Ronan et al. (2020)	Children (2668)	9–11	Cross-sectional study Observational data Mediation analysis	(1) Increased BMI was associated with lower executive function. Reduced thickness in the rostral medial and superior frontal cortex, the inferior frontal gyrus, and the lateral orbitofrontal cortex partially accounted for reductions in executive function. (2) These results suggest that childhood obesity is associated with compromised executive function. This relationship may be partly explained by BMI-associated reduced cortical thickness in the PFC.
Jiang et al. (2023)	Obesity (258): 125F/133M; Normal weight (266): 125F/141M	9–10	Longitudinal observational study	(1) Significant group × time effects on GM volume were observed in the prefrontal lobe, thalamus, right precentral gyrus, caudate, and parahippocampalgyrus/amygdala between OB and NW. (2) Children with obesity had significantly lower regional gray matter in widespread brain regions which are important for functions such as motor control and working memory, compared with the children with normal weight had greater reductions in GM volume in these regions over the 2-year period. Body mass index (BMI)was negatively correlated with GM volume in prefrontal lobe and with matrix reasoning ability at baseline and 2-year follow-up. (3) In children with obesity, Picture Test was positively correlated with GM volume in the left orbital region of the inferior frontal gyrus (OFCinf_L) at baseline and was negatively correlated with reductions in OFCinf_L volume (2-year follow-up vs. baseline).
Mestre et al. (2020)	Children (102): 54F/48M	12–18	Cross-sectional population-based study	In adolescents, greater body weight is associated with altered hippocampal tissue integrity but not altered volumes.
Steegers et al. (2021)	Children (3160): 1589F/1571M	9–11	Cross-sectional magnetic resonance study	In adolescents, cortical thickness is positively associated with BMI.
Zhang et al. (2023)	Obesity/Overweight (232); Normal weight (244)	11–12	Cross-sectional case–control study	Compared with NW, OW/OB showed smaller GM volumes in the dorsal anterior cingulate cortex (dACC), medial orbital frontal cortex (mOFC_L/R), medial superior frontal gyrus (mSFG_L/R), and left superior frontal gyrus.
de Groot et al. (2017)	Obesity (23); Lean (19)	12–16	Cross-sectional observational study	The adolescents with obesity have greater pallidum volume compared with the lean adolescents and show a positive correlation between pallidum volume and executive performance, independent of presence of metabolic syndrome.
Perlaki et al. (2018)	Children (51); 32F/19M	10–16	Cross-sectional observational study	Childhood obesity is associated with enlarged structural volumes, but decreased GM density in the reward system.
Ross et al. (2015)	Obesity (79); Non 0besity (51)	17–22	Cross-sectional observational study	Adolescents with obesity had significant reductions in OFC thickness, but the groups did not differ on OFC volume, ACC volume, ACC cortical thickness, or global brain atrophy.
Moreno-López et al. (2012)	Excess weight (36); Normal weight (16)	12–17	Cross-sectional observational study	The excess weight adolescents had increased right hippocampal GM regional volumes compared to lean controls.
Brady et al. (2025)	The type 2 diabetes (20); Obesity (19)	13–20	Cross-sectional case–control study	A significant and positive relationship between CBF (perfusion) and GMV (structure) among youth with obesity, with and without T2D, was demonstrated in several clusters encompassing the calcarine, cuneus, precuneus, occipital, fusiform, lingual, right insula, and right precentral brain regions.
Ou et al. (2015)	Obesity (12); Normal weight (12)	8–10	Cross-sectional case–control study	(1) Children with obesity had significantly lower regional gray matter in widespread brain regions which are important for functions such as motor control and working memory. (2) Compared with normal weight children, children with obesity had significant, family wise error corrected regional gray matter reduction in the right middle temporal gyrus, left and right thalami, left superior parietal gyrus, left pre/postcentral gyri, and left cerebellum.
Aytaç Kaplan et al. (2025)	Obesity (79): 55F/24M; Normal weight (67): 42F/25M	6–18	Cross-sectional observational study	In the obesity group, right olfactory bulb area and right olfactory bulb volume were significantly higher compared to the other groups, while left olfactory bulb area was higher in both the obesity and obesity groups.
Tirsi et al. (2013)	Obesity (62); Normal weight (40)	10–20	Cross-sectional case–control study	Adolescents with obesity had greater global cerebral atrophy (cc) and smaller hippocampal volumes (cc).
Nouwen et al. (2017)	Type 2 diabetes (15) Obesity (21); Normal weight (22)	12–18	Cross-sectional case–control study	Adolescents with type 2 diabetes and adolescents with obesity had reduced gray matter volume in the right hippocampus, left Putamen and caudate, bilateral amygdala and left thalamus compared to healthy weight controls.
Yokum et al. (2012)	Obesity (17); Lean (31); Overweight (36)	Mean = 18	Prospective cohort study	Participants with obesity had less total GM volume than lean and participants with overweight. Trend-level reduced GM volumes in the superior frontal gyrus and middle frontal gyrus were related to increases in BMI over 1-year follow-up.
Karaoglan and Colakoglu Er (2020)	Overweight (31); Obesity (32); Severe obesity (43); Normal weight (89)	6–18	Cross-sectional observational study	Mean OBV was higher in children with obesity than in those of normal weights. The means of OBV are found higher in the children with overweight and obesity than in those of obesity.
Cheng et al. (2023)	Obesity/Overweight (101): *F* (12)/M (89)	3–12	Cross-sectional observational study	Overweight/obesity positively correlated with SRS-2 total points; gray matter volume in the left dorsolateral superior frontal gyrus (Frontal_Sup_L GMV) negatively correlated with SRS-2 total points; and overweight/obesity negatively correlated with Frontal_Sup_L GMV.

MRI, magnetic resonance imaging; ACC, anterior cingulate cortex; BMI, body mass index; CBF, cerebral blood flow; CC, corpus callosum; F, female; GM, gray matter; GMV, gray matter volume; L, left; M, male; NW, normal weight; OBV, olfactory bulb volume; OB, obesity; OFC, orbitofrontal cortex; OV, overweight; PFC, prefrontal cortex; R, right; SRS, social responsiveness scale.

### 3.4 Structural MRI-white matter

In terms of structural MRI-white matter, there were 9 studies involved. Among these studies, two were purely cross-sectional studies, 3 were cross-sectional observational studies, 3 were cross-sectional case–control studies, and one was a prospective study.

Most studies consistently indicate impaired white matter integrity in adolescents with obesity, characterized primarily by reduced FA. Multiple clusters of reduced FA in key fiber tracts such as the left brainstem, right optic radiation, left internal capsule, and left splenium of the corpus callosum were observed in adolescents with obesity (Yau et al., 2014). Gender-specific patterns were also noted: females showed decreased integrity in the corticospinal tract and uncinate fasciculus, while males showed it primarily in the superior frontal corpus callosum and left inferior fronto-occipital fasciculus (Carbine et al., 2020). Similar reductions in FA were observed in the uncinate fasciculus and commissural tracts in other populations, sometimes accompanied by decreased vascularization in the occipital lobe (Samoilova et al., 2022). Longitudinal evidence supports this trend, and higher baseline BMI z-scores were negatively correlated with neurite density and FA values in extensive white matter regions (e.g., bilateral inferior fronto-occipital fasciculus, anterior thalamic radiation, striato-frontal pathway) and positively correlated with radial diffusivity (Kaltenhauser et al., 2023). Moreover, reduced area of the anterior corpus callosum was linked to slower cognitive processing speed in adolescents with obesity (Sweat et al., 2017).

It should be noted that impact of obesity on white matter is not unidirectional. Some studies observed increased FA values (Ou et al., 2015) or white matter volume (Yokum et al., 2012) in specific regions. Interestingly, physical fitness appears to play a protective or modulating role, with muscular fitness showing associations with white matter microstructure specifically in frontal areas (Rodriguez-Ayllon et al., 2020) and reports exist of enhanced integrity in certain fiber tracts in males with obesity (Carbine et al., 2020). Other changes included reduced white matter intensity in the lateral occipital cortex (Brooks et al., 2023), and increased intensity in the prefrontal lobe, and reduced brain parenchymal volume (Cazettes et al., 2011). For more details see Table 5.

**Table 5:** Structural MRI-White matter studied investigating brain functions in children or adolescents.

Study	Participants		Design	Main findings
	Subjects (*n*)	Age (years old)		
Yau et al. (2014)	Obesity (30); Lean (30)	14–20	Case–control study with a cross-sectional design	In the absence of gross brain volume differences or clinically significant WM hyperintensities on the FLAIR image, the VANCOVA analysis of the FA maps identified seven significant clusters, six of which showed age-adjusted WM FA reductions among adolescents with obesity. The clusters showing FA reduction were located, by order of size, in the left temporal stem, right optic radiation, left internal capsule, left splenium, left external capsule, and left optic radiation.
Sweat et al. (2017)	Obesity (108); Healthy weight (54)	17–22	Cross-sectional neuroimaging study	(1) We also found differences between the weight groups on the area of the anterior portion of the CC, but not the overall CC. (2) Only the controlled oral word association test (COWAT) was significantly correlated with the area of the anterior portion of the CC.
Brooks et al. (2023)	Overweight (736); Obesity (672); Normal BMI (3514)	8–11	Cross-sectional magnetic resonance study	(1) Lower white matter intensity was estimated bilaterally in lateral occipital cortex, which may be correlated with aberrant connectedness in these areas, and is also coupled to both the DM and frontoparietal control networks in perception. (2) Higher white matter intensity was estimated in the right anterior prefrontal cortex of participants with obesity but not overweight.
Carbine et al., 2020	Obesity/Overweight (40); Normal weight (47)	12–20	Cross-sectional case–control study	(1) We found decreased white matter integrity, as indicated by decreased FA values, in OV/OB female adolescents in the left corticospinal tract and the left and right uncinate tracts. (2) We also found decreased white matter integrity in OV/OB male adolescents in the superior frontal portion of the corpus callosum and left inferior fronto-occipital fasciculus. (3) The right inferior fronto-occipital fasciculus, the left cingulum, left corticospinal tract, and the orbital frontal corpus callosum in OV/OB males and the anterior frontal corpus callosum in both males and females showed increased white matter integrity in OV/OB adolescents relative to NW adolescents. These results were also generally supported by the post-hoc MD, AD, and RD analyses.
Rodriguez-Ayllon et al. (2020)	Children (104): 43F/61M	8–11	Cross-sectional observational study	Using the voxel-wise approach, we identified a small cluster in the left lateral frontal lobe where children with greater upper-body muscular fitness showed higher FA.
Kaltenhauser et al. (2023)	Underweight (191); Overweight (683); Obesity (656); Healthy weight (3046)	9–10	An observational study with cross-sectional and longitudinal analyses.	(1) Higher baseline BMIz scores were negatively associated with interval changes in ND and FA of bilateral inferior-fronto-occipital fasciculi, anterior thalamic radiations, striatal inferior frontal cortices, and corpus callosum, including forceps minor, ND of the right cingulate gyrus. (2) Higher BMI z scores at baseline were positively associated with interval changes in RD of bilateral inferior-fronto-occipital fasciculi, left striatal inferior frontal cortex, and right anterior thalamicradiation.
Cazettes et al. (2011)	Obesity (48); Lean (31)	14–21	Cross-sectional observational study	Intracranial vault volume did not differ between groups, but adolescents with obesity had smaller intracranial vault-adjusted brain parenchymal volumes.
Ou et al. (2015)	Obesity (12); Normal weight (12);	8–10	Cross-sectional case–control study	Children with obesity also had higher white matter in multiple regions in the brain and higher DTI measured fractional anisotropy (FA) values in part of the left-brain association and projection fibers.
Yokum et al. (2012)	Obesity (17); Overweight (36); Lean (31)	Mean = 18	Prospective cohort study	Participants with obesity had lower total WM volume than participants with overweight. BMI correlated with higher WM volumes in the middle temporal gyrus, fusiform gyrus, parahippocampal gyrus, rolandic operculum and dorsal striatum.

MRI, magnetic resonance imaging; AD, axial diffusivity; BMI, body mass index; FLAIR, fluid-attenuated inversion recovery; VANCOVA, voxel-wise analysis of covariance; CC, corpus callosum; DM, default-mode; DTI, diffusion tensor imaging; F, female; M, male; FA, fractional anisotropy; MD, mean diffusivity; ND, neurite density; NW, normal weight; OB, obesity; OV, overweight; RD, radial diffusivity; WM, white matter.

### 3.5 Quality assessment results

According to the quality assessment scale, the included literature generally demonstrated high research quality, supporting the robustness of this review’s conclusions (Table 6). In the dimension of directness, 41 studies were rated as “high quality,” indicating that most studies were designed to directly test differences in brain structure/function related to obesity in children/adolescents. Four studies were of “adequate quality,” meaning they lacked relevance in participant selection or their outcome measures diverged from the review’s core focus. Regarding precision, 35 studies showed “high quality,” mostly possessing medium-to-large sample sizes or rigorous longitudinal designs, ensuring statistical power. Ten studies were of “adequate quality” because the sample size did not meet the expected standard. Regarding bias control, 42 studies were rated “high quality,” reflecting methodological rigor in participant recruitment, exposure standardization, outcome measurement, and effective correction for confounders like age, gender, and socioeconomic status. Three studies were of “adequate quality,” meaning confounding factors and missing data were not adequately controlled.

**Table 6:** Study details and quality assessment results.

Author(s), year	Reference	Absence of bias	Directness	Precision
Resting-state fMRI				
Moreno-Lopez et al. (2016)	Neuroimage Clin: 12: 262–268	++	++	++
Grigorian et al. (2022)	Int J Neuropsychopharmacol; 25(6): 448–456.	++	+	++
Liu et al. (2025)	Pediatric Research; s41390-025-03995-1	++	++	++
Martín-Pérez et al. (2019)	J Am Acad Child Adolesc Psychiatry; 58(2): 211-220.e5	++	++	++
Pujol et al. (2021)	Cereb Cortex; 31(9): 4376–4,385	++	++	++
Brooks et al. (2023)	Int J Obes (Lond); 47(7): 590–605.	++	++	++
Task-based fMRI				
Brooks et al. (2023)	Int J Obes (Lond); 47(7): 590–605.	++	++	++
Bohon (2017)	PLoS One; 12(2): e0172604	++	++	+
Moreno-Padilla et al., 2018	Appetite; 1:131:7–13	+	++	++
Rapuano et al. (2016)	Cereb Cortex; 26(6): 2602–2,611	++	++	+
Verdejo-García et al. (2015)	Hum Brain Mapp; 36(1): 226–237	++	++	++
Sewaybricker et al. (2019)	PediatrObes; 14(2): e12486	++	++	+
Boutelle et al. (2015)	Int J Obes (Lond); 39(4): 620–8	+	++	+
Bruce et al. (2010)	Int J Obes (Lond); 34(10): 1494–500	++	++	+
Davids et al. (2010)	Int J Obes (Lond); 34(1): 94–104	++	++	++
Bruce et al. (2013)	J Pediatr; 162(4): 759-764.e2	++	++	+
Mestre et al. (2017)	Int J Obes (Lond); 41(10): 1496–1,502	++	++	+
Delgado-Rico et al. (2013)	Obesity (Silver Spring); 21(8): 1662–8	++	++	++
Kerem et al. (2022)	J Clin Child Adolesc Psychol; 51(5): 701–714	++	+	+
Structural MRI-gray matter				
Ronan et al. (2020)	Cereb Cortex. 2020 Apr 14; 30(4): 2519–2,528	++	++	++
Jiang et al. (2023)	Cereb Cortex. 2023 Mar 21; 33(7): 3674–3,682	++	++	++
Mestre et al. (2020)	Obesity (Silver Spring). 2020 Jul; 28(7): 1325–1,331	++	++	++
Steegers et al. (2021)	Brain Struct Funct. 2021 Apr; 226(3): 787–800	++	++	++
Zhang et al. (2023)	Cereb Cortex. 2023 May 9; 33(10): 6335–6,344	++	++	++
de Groot et al. (2017)	Pediatr Obes. 2017 Aug; 12(4): e33-e36	+	++	++
Perlaki et al. (2018)	PLoS One. 2018 Oct 18; 13(10): e0205331	++	++	++
Ross et al. (2015)	Appetite. 2015 Oct; 93:44–50	++	+	++
Moreno-López et al. (2012)	PLoS One. 2012; 7(11): e49185	++	++	++
Brady et al. (2025)	Neuroimage Clin. 2025 Jun 18; 47: 103828	++	++	++
Ou et al. (2015)	J Magn Reson Imaging. 2015 Nov; 42(5): 1205–13	++	++	+
Aytaç Kaplan et al. (2025)	Children (Basel). 2025 Jul 31; 12(8):1009	++	++	++
Tirsi et al. (2013)	Obesity (Silver Spring)	++	++	++
Nouwen et al. (2017)	Neuroimage Clin. 2017 Jul 5; 16: 43–51	++	++	++
Yokum et al. (2012)	Int J Obes (Lond). 2012 May; 36(5): 656–64	++	++	++
Karaoglan and Colakoglu Er (2020)	Int J Pediatr Otorhinolaryngol. 2020 Dec; 139: 110415	++	++	++
Cheng et al. (2023)	Brain Sci. 2023 Jan 21; 13(2): 180	++	++	++
Structural MRI-white matter				
Yau et al. (2014)	Obesity (Silver Spring). 2014 Aug; 22(8): 1865–71	++	++	++
Sweat et al. (2017)	Child Obes. 2017 Jun; 13(3): 190–196	++	++	++
Brooks et al. (2023)	Int J Obes (Lond). 2023 Jul; 47(7): 590–605	++	++	++
Carbine et al., 2020	Brain Imaging Behav. 2020 Feb; 14(1): 308–319	++	++	++
Rodriguez-Ayllon et al. (2020)	Sci Rep. 2020 Jul 27; 10(1): 12469	++	+	++
Kaltenhauser et al. (2023)	JAMA Netw Open. 2023 May 1; 6(5): e2314193	++	++	++
Ou et al. (2015)	J Magn Reson Imaging. 2015 Nov; 42(5): 1205–13	++	++	+
Yokum et al. (2012)	Int J Obes (Lond). 2012 May; 36(5): 656–64	++	++	++
Cazettes et al. (2011)	AJNR Am J Neuroradiol. 2011 Dec; 32(11): 2037–42	++	++	++

++High quality; +Denotes adequate quality.

## 4 Discussion

**Figure 2:**
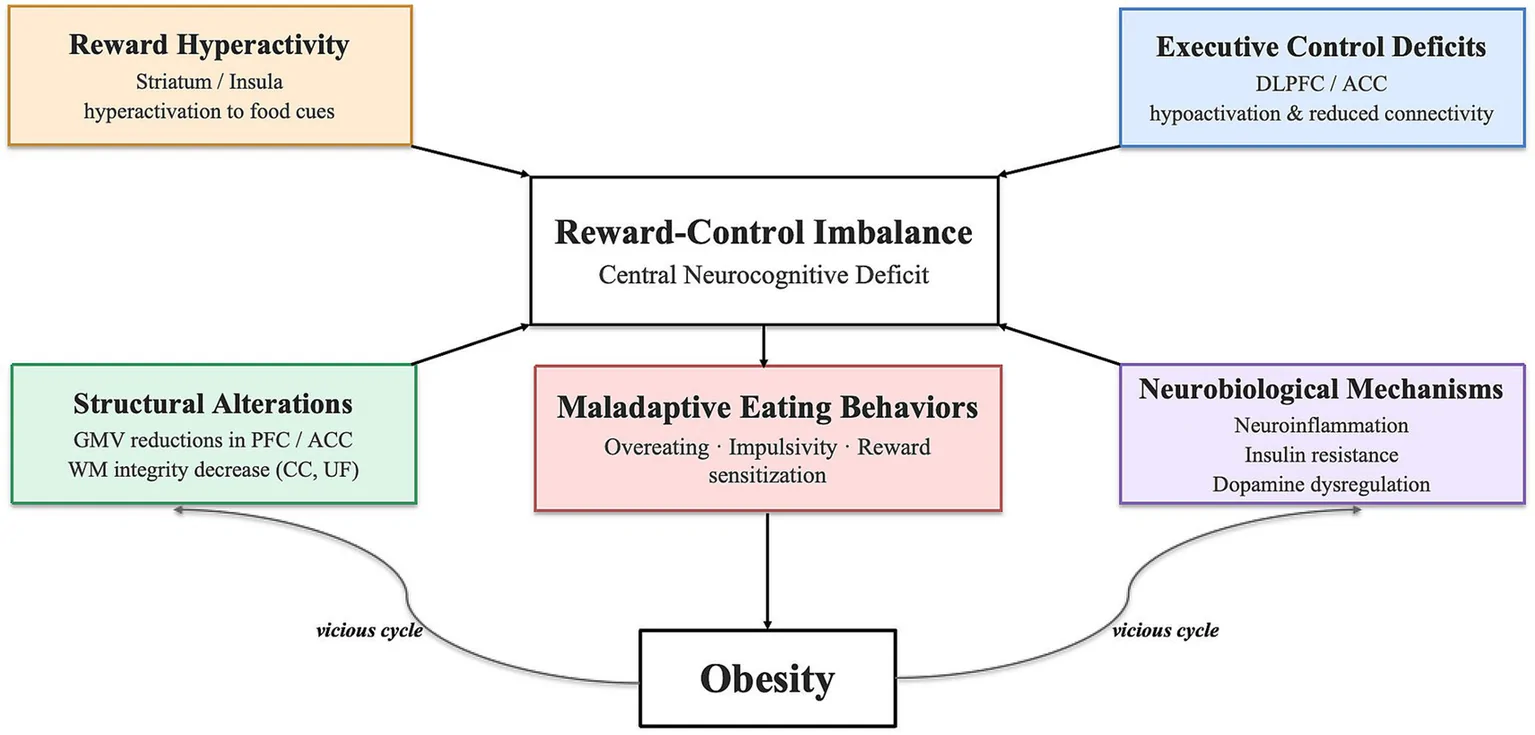
Integrative framework of obesity-related brain alterations in children and adolescents in this review. DLPFC, dorsal lateral prefrontal cortex; ACC, anterior cingulate cortex; GMV, gray matter volume; WM, white matter; PFC, prefrontal cortex; CC, corpus callosum; UF, uncinate fasciculus.

Synthesizing the convergent brain structural and functional findings reviewed above, we proposed an integrative conceptual framework to distill the complex interplay among neural phenotypes in childhood and adolescent obesity (Figure 2).

### 4.1 Resting-state fMRI

Children and adolescents with obesity are at increased risk for psychiatric comorbidities, including ADHD, depressive disorders, and anxiety disorders, which may further influence brain structure and function. One study reported that approximately 30% of children and adolescents with overweight status or obesity presenting for weight management met criteria for at least one mental health disorder, with ADHD and depressive disorders being the most common (Galler et al., 2024). Similarly, another study found that psychiatric comorbidity was present in a substantial proportion of youth with obesity, with internalizing disorders (anxiety, depression) being more prevalent in older adolescents and externalizing disorders (ADHD, disruptive behaviors) more common in younger children (Adıgüzel Akman et al., 2025). These findings suggest that the neuroimaging abnormalities observed in pediatric obesity may not be solely attributable to metabolic factors but may also reflect underlying psychiatric vulnerabilities or the neurobiological sequelae of chronic psychiatric symptoms. Conversely, obesity-related brain alterations in reward and executive control networks may predispose individuals to the development of psychiatric symptoms, creating a bidirectional relationship that warrants further investigation. The coexistence of psychiatric comorbidities highlights the need for integrated, multidisciplinary assessment and intervention approaches for children and adolescents with obesity.

An important limitation of the current literature, and consequently of this review, is the broad age range across studies, which often combines children and adolescents into a single analytical group. Brain maturation is a dynamic and protracted process: early childhood is characterized by rapid synaptic proliferation and gray matter expansion; middle childhood involves synaptic pruning and cortical thinning; puberty marks a period of significant white matter myelination and functional network reorganization; and late adolescence is associated with the refinement of executive control networks. These developmental stages are likely to differentially modulate the impact of obesity on brain structure and function (Sadler et al., 2023). For instance, exposure to metabolic and inflammatory insults during early critical periods may have more profound and lasting effects on brain development than similar exposure during later stages. Conversely, the heightened reward sensitivity and immature prefrontal control during puberty may render adolescents particularly vulnerable to obesity-related functional dysregulation in reward and executive networks. Unfortunately, the majority of studies included in this review did not stratify their analyses by developmental stage or pubertal status, precluding a systematic assessment of age-related effects. Future studies with larger samples should specifically examine how developmental stage moderates the relationship between obesity and neuroimaging outcomes, and we recommend that future reviews adopt developmental stratification as an analytical framework.

The most consistent finding regarding resting-state fMRI in children and adolescents with obesity is the disruption of intrinsic interactions between large-scale brain networks, particularly the weakening or loss of negative coupling between the DMN and the ECN.

Children and adolescents with obesity exhibit declines in cognitive function, attention, inhibitory control, and self-regulation ability, which may be related to decreased FC between the DMN and the prefrontal-striatal network, especially weakened synchrony in the dorsal prefrontal striatum. Additionally, connectivity in the prefrontal-parietal network involved in cognitive control is reduced (Tomasi and Volkow, 2024), suggesting impaired executive function. This aligns with our research findings. The PFC is the core region of executive function, and its anatomy, neurotransmitters, and network properties determine its dominant role in higher-level cognitive control. Obesity is associated with elevated serum leptin and insulin levels, and decreased receptor sensitivity impairs the prefrontal cortex’s inhibitory function on satiety signals. Leptin/insulin signaling regulates GABA-mediated inhibition in the hypothalamus-prefrontal circuit (Volkow et al., 2011), resistance weakens this inhibition, leading to weakened negative coupling between the ECN and DMN. In a normal brain performing cognitive tasks, the ECN activates to maintain external attention, while the DMN (responsible for introspection and self-reference) is inhibited, presenting a dynamic “push-pull” antagonistic relationship. This review found that this negative coupling is significantly weakened in adolescents with obesity. This may reflect a reduced ability of the brain to effectively switch from “self-immersion mode” to “external control mode,” which could contribute to inefficient allocation of cognitive resources, manifesting as difficulty concentrating and decreased self-regulation ability in some individuals.

Furthermore, obesity is accompanied by systemic low-grade inflammation; pro-inflammatory cytokines (IL-6, TNF-*α*) can cross the blood–brain barrier and activate microglia in the hypothalamus and PFC. Activated microglia release ROS and cytokines, destroying synaptic plasticity, especially in long-range white matter fibers (key pathways connecting ECN and DMN). Moreover, obesity affects relevant vascular endothelial function, thereby reducing local blood flow regulation and affecting the reliability of the blood-oxygen-level-dependent signals (Grigorian et al., 2022), particularly in high-metabolic regions of the DMN (posterior cingulate cortex, medial PFC). Restricted blood flow regulation further weakens ECN-DMN synchronization waveforms, leading to decreased communication efficiency between networks.

Multiple rs-fMRI studies also indicate that obesity is accompanied by decreased Dopamine D2 receptor availability in the NAc or ventral tegmental area, enhancing reward-driven ascending signals (Volkow et al., 2011). Decreased dopamine D2 receptor availability increases reward sensitivity to high-calorie foods and inhibits the executive control network’s suppression of the DMN, further weakening the negative interaction between the two networks. When ECN-DMN negative regulation fails, the reward network responds more strongly to food stimuli (enhanced connection between DMN and salience network), leading to increased attentional capture by high-calorie foods and increased frequency of impulsive eating. These effects superimpose, impairing the negative regulation function of the ECN on the DMN, manifesting as declines in attention, impulse inhibition, and self-regulation. This neurobiological framework provides clear targets for obesity interventions (e.g., weight loss, exercise, anti-inflammatory treatments, improving insulin/leptin sensitivity).

### 4.2 Task-based fMRI

Unlike resting-state MRI, which reflects “intrinsic network architecture,” task-based fMRI reveals a “dual systems model” imbalance in the obesity brain when facing specific stimuli (e.g., high-calorie food, decision-making tasks): specifically, the coexistence of high reactivity in subcortical reward systems and low reactivity in cortical control systems.

When facing food cues (especially high-calorie ones), both children and adolescents with obesity exhibit a significant imbalance between high reactivity of the reward system and low reactivity of cognitive control and interoceptive systems; this change in neural activity patterns leads directly to inhibitory control failure and impulsive eating behaviors. Specifically, upon exposure to high-calorie food stimuli, reward networks (e.g., striatum, NAc) in adolescents with obesity show overactivation (Bruce et al., 2010; Rapuano et al., 2016), while inhibitory signals from the PFC responsible for decision-making and inhibition are significantly weakened (Bruce et al., 2013). This dysregulation of “gas pedal” and “brake” may be related to pathological changes in dopamine reward pathways.

Under high-calorie food intake or visual stimulation, the dopamine pathway from the ventral tegmental area to the NAc is strongly activated. However, reduced availability of dopamine D2 receptors in the brains of individuals with obesity is associated with an elevated reward threshold. Consequently, to achieve equivalent pleasure, the brain requires stronger external stimuli (i.e., more high-calorie food), manifested in task-based fMRI as hyperactivation of the striatum to food cues (Boutelle et al., 2015). Simultaneously, this intense reward-driven signal overwhelms the prefrontal cortex’s control ability, leading to weakened FC in the prefrontal-striatal circuit, making top-down inhibitory control difficult to achieve. This pathological “craving” signal manifests as intense activation of reward brain regions in task-state imaging (Moreno-Padilla et al., 2018). Meanwhile, the dorsolateral prefrontal cortex and ACC, responsible for inhibitory control, show insufficient activation during inhibition tasks (Verdejo-García et al., 2015). Neurobiologically, this may be related to central insulin/leptin resistance. Under normal conditions, these hormones act on the hypothalamus-prefrontal circuit to enhance inhibitory signals; in a state of resistance, top-down inhibition from the PFC to the reward circuit is weakened.

Additionally, tasking-state studies reveal neural deficits in interoception and emotional regulation in adolescents with obesity (Boutelle et al., 2015). Previous research found that during food induction, activation of the insula—closely related to interoception (such as satiety signal processing)—is weakened. This suggests that the brains of children with obesity may fail to effectively integrate peripheral satiety signals (e.g., leptin, insulin), resulting in a lack of physiological termination signals for eating behaviors. Concurrently, FC between the amygdala and PFC is significantly reduced, weakening the emotional regulation network’s buffering effect on impulses.

The neuroimaging findings reviewed here have several potential clinical implications. First, the consistent evidence of prefrontal structural and functional alterations suggests that early screening for executive function deficits—particularly inhibitory control and working memory—may be valuable in children at risk for obesity, as these cognitive vulnerabilities could be targeted before they become entrenched. Second, the association between obesity and psychiatric comorbidities (e.g., ADHD, depression, anxiety) supports the need for integrated, multidisciplinary obesity management that addresses both metabolic and mental health dimensions. Third, family-based interventions that target both dietary behaviors and family-level emotional regulation may be particularly effective given the involvement of reward and emotion regulation circuits in obesity. Fourth, executive function training programs (e.g., computerized cognitive training, physical exercise programs that engage prefrontal circuits) may serve as adjunctive interventions to enhance cognitive control and support weight management. Finally, the developmental sensitivity of the pubertal period highlights the importance of prevention strategies that target this critical window, such as school-based programs that promote healthy eating and physical activity while also fostering emotion regulation and impulse control skills.

The included studies exhibit considerable methodological heterogeneity across multiple dimensions. Participant age ranges vary widely, with some studies including children as young as 6 years and others focusing exclusively on adolescents up to 18 years, yet few studies stratify by age or examine age × obesity interactions. Obesity definitions also vary: some studies use age- and sex-specific BMI percentiles, others use BMI z-scores, and still others use absolute BMI thresholds. MRI acquisition parameters differ across studies (e.g., scanner field strength, TR, TE, voxel size), as do preprocessing pipelines (e.g., software packages, motion correction strategies, denoising approaches, smoothing parameters). Cognitive paradigms in task-based fMRI studies vary substantially across inhibitory control tasks, decision-making tasks, and food cue paradigms, making direct comparisons challenging. Statistical approaches also differ, with some studies using whole-brain analyses, others using ROI-based approaches, and still others using network-based analyses. This heterogeneity may contribute to the variability in findings across studies and limits the robustness of the overall conclusions. We recommend that future studies adopt standardized protocols, such as those from the ABCD Study or ENIGMA consortium, to facilitate cross-study comparability.

Executive function is a multidimensional construct that encompasses several distinct but interrelated cognitive domains, including inhibitory control (the ability to suppress prepotent responses), working memory (the capacity to maintain and manipulate information online), cognitive flexibility (the ability to shift between tasks or mental sets), planning (the capacity to organize behavior toward future goals), and decision-making (the ability to evaluate options and select optimal courses of action). The neuroimaging evidence reviewed here supports domain-specific effects of obesity. Inhibitory control deficits appear to be the most consistently documented domain, with studies reporting reduced prefrontal activation during response inhibition tasks and impaired prefrontal-striatal connectivity. Working memory deficits are also supported by structural MRI findings showing reduced gray matter volume in the dorsolateral prefrontal cortex and by task-based fMRI studies showing altered prefrontal activation during working memory tasks. Evidence for cognitive flexibility deficits is more limited but is supported by DTI findings of reduced white matter integrity in the cingulum bundle and inferior fronto-occipital fasciculus, which connect prefrontal regions involved in set-shifting. Decision-making impairments are supported by task-based fMRI studies showing abnormal insula and midbrain activation during risk tasks. The specificity of these domain-selective associations suggests that obesity does not impair executive function globally but rather affects distinct cognitive and neural systems differentially.

In summary, striatal overactivation, weakened prefrontal inhibition signals, and insula/amygdala functional abnormalities observed in the task state collectively construct an “impulse-reward-loss of control” neural vicious cycle. This neural mechanism may help explain why adolescents with obesity, when facing food temptations, not only generate stronger desires to eat (reward sensitization) but also struggle to resist temptation through cognitive control (impaired inhibition), potentially accompanied by blunted satiety (interoceptive deficit), thereby exacerbating obesity development. This also suggests that future intervention strategies need to focus not only on enhancing cognitive control (e.g., prefrontal training) but also on how to reshape reward system sensitivity through behavioral or pharmacological means.

### 4.3 Structural MRI-gray matter

Executive function is a multidimensional construct encompassing several distinct but interrelated cognitive domains, including inhibitory control (Xu et al., 2023) (the ability to suppress prepotent responses), working memory (the capacity to maintain and manipulate information online), cognitive flexibility (the ability to shift between tasks or mental sets), planning (the capacity to organize behavior toward future goals), and decision-making (the ability to evaluate options and select optimal courses of action). The neuroimaging evidence reviewed here supports domain-specific effects of obesity. Inhibitory control deficits appear to be the most consistently documented domain, with studies reporting reduced prefrontal activation during response inhibition tasks and impaired prefrontal-striatal connectivity (Ronan et al., 2020). Working memory deficits are supported by structural MRI findings showing reduced gray matter volume in the dorsolateral prefrontal cortex and by task-based fMRI studies showing altered prefrontal activation during working memory tasks. Evidence for cognitive flexibility deficits is more limited but is supported by DTI findings of reduced white matter integrity in the cingulum bundle and inferior fronto-occipital fasciculus, which connect prefrontal regions involved in set-shifting (Brooks et al., 2023). Decision-making impairments are supported by task-based fMRI studies showing abnormal insula and midbrain activation during risk tasks. The specificity of these domain-selective associations suggests that obesity does not impair executive function globally but rather affects distinct cognitive and neural systems differentially.

Echoing the “dual system” functional imbalance revealed by task-fMRI, structural MRI further confirms a significant “structural dissociation pattern” at the anatomical level in the obesity brain: the coexistence of atrophy in cortical regions responsible for cognitive control and hyperplasia in subcortical regions responsible for reward drive.

In terms of control area damage and drive area compensation, both children and adolescents with obesity exhibit distinct features of “control area damage” and “drive area compensation” in gray matter structure. Specifically, individuals in a long-term obesity state generally show reduced gray matter volume or cortical thinning in the PFC, ACC, and OFC (Xu et al., 2024). In sharp contrast, subcortical structures closely related to reward processing and emotional memory, such as the globus pallidus, amygdala, and hippocampus, tend to show increased volume. This “trade-off” structural remodeling provides a macro-anatomical basis for the “gas pedal stuck, brakes failed” phenomenon observed at the functional level, directly limiting executive function and reinforcing reward-seeking behavior.

In terms of vicious cycle of inflammation-insulin resistance, from a neurobiological perspective, prefrontal atrophy profoundly reflects the vicious cycle of “neuroinflammation-insulin resistance” (Boutelle et al., 2015). High-fat diet-induced systemic inflammation can activate central microglia, promoting the release of pro-inflammatory cytokines such as TNF-*α* and IL-6 (Thaler et al., 2012). These molecules have significant neurotoxicity, capable of directly destroying synaptic plasticity, inhibiting neurogenesis, and inducing neuronal apoptosis, ultimately leading to gray matter loss. Simultaneously, insulin sensitivity in the brains of individuals with obesity (especially the prefrontal lobe) is reduced. Since insulin signaling pathways are crucial for maintaining the neuronal cytoskeleton, synaptic density, and energy metabolism, the impairment of this signal under resistance directly deprives prefrontal neurons of nutritional support, exacerbating cortical atrophy. Conversely, the increased volume of subcortical reward regions may reflect increased dendritic spine density and synaptic remodeling caused by long-term high-calorie food stimulation, a pathological structural compensation similar to addiction mechanisms.

In summary, prefrontal atrophy and limbic system hyperplasia collectively build an anatomical foundation of “impaired control hardware - reinforced drive hardware.” This structural change may contribute to why adolescents with obesity show reduced capacity for top-down inhibitory control when facing temptation, as prefrontal structural integrity is a critical determinant of executive function performance. This pattern is consistent with the finding that prefrontal cortical thinning mediates the relationship between obesity and declined executive function (Thaler et al., 2012). This also suggests that future intervention strategies (such as exercise or medication) must commit to improving the brain metabolic environment (e.g., raising BDNF levels, alleviating insulin resistance) to reverse this structural damage and promote neural regeneration and repair in key brain regions.

### 4.4 Structural MRI-white matter

Unlike the “regional structural changes” reflected by gray matter, DTI reveals a “connectivity integrity collapse” at the whole-brain network communication level in the obesity brain: damage to the microstructure of key fiber tracts is associated with a comprehensive decline in information transmission efficiency between brain regions.

In terms of network communication blockage, both children and adolescents with obesity exhibit widespread declines in integrity in white matter microstructure; this blockage of network communication directly weakens the collaborative capability among brain systems. Studies consistently find that in key pathways connecting the prefrontal lobe and the limbic system (such as the corpus callosum, uncinate fasciculus, inferior fronto-occipital fasciculus), individuals with obesity universally exhibit significantly reduced FA. This indicates reduced myelination or disordered axonal arrangement, making it difficult for inhibitory signals from the prefrontal lobe to be transmitted quickly and accurately to the reward center, resulting in the attenuation or loss of “top-down” control signals during transmission.

In terms of oligodendrocyte damage and developmental deviation, the mechanism of this widespread white matter damage involves the inflammatory susceptibility of oligodendrocytes and deviations in developmental trajectories. First, as obesity is a state of systemic low-grade chronic inflammation, circulating inflammatory factors can damage the blood–brain barrier and mediate microglial activation (Thaler et al., 2012), which then attacks oligodendrocytes responsible for myelin formation, hindering myelin formation and repair. Second, leptin resistance weakens its neurotrophic role in white matter fibers (McGregor and Harvey, 2018). Furthermore, the abnormally high FA values observed in specific brain regions in some studies may reflect “deviation from developmental trajectory,” meaning that during the critical remodeling period of adolescence, the brain exhibits pathological “premature maturation” or insufficient synaptic pruning. This abnormal structural solidification limits the plasticity of neural networks, causing the brain to lock prematurely into an inefficient connectivity mode.

In summary, the impaired white matter integrity and developmental trajectory deviations observed via DTI collectively construct a neuropathological environment of “network communication blockage-restricted plasticity.” This mechanism may help explain why obesity is associated not only with functional abnormalities of local brain regions but also with a decline in whole-brain network integration capability, leading to poor performance in complex tasks involving multi-region synergy (such as emotional regulation, impulse inhibition). This also suggests that future intervention strategies need to pay special attention to the critical window of puberty, protecting vulnerable oligodendrocytes through early anti-inflammatory and metabolic interventions to prevent permanent deviation of brain network development trajectories.

## 5 Limitations and future directions

A critical limitation of the current literature is that the majority of studies are cross-sectional, precluding causal inferences about the direction of the relationship between obesity and brain alterations. While the “obesity → brain” pathway is biologically plausible—given that obesity induces neuroinflammation, insulin resistance, and altered reward signaling—the reverse pathway is equally plausible: pre-existing differences in executive function, impulse control, reward sensitivity, or psychiatric symptoms may increase susceptibility to weight gain and obesity. For example, individuals with reduced prefrontal inhibitory control may find it more difficult to resist high-calorie food cues, leading to weight gain over time. Similarly, heightened reward sensitivity may increase the reinforcing value of food, contributing to overeating. Thus, the neuroimaging differences observed between children and adolescents with obesity and their normal-weight peers may reflect a combination of obesity-induced brain changes, pre-existing neurobiological vulnerabilities, and bidirectional interactions. To disentangle these pathways, future research must prioritize longitudinal designs that track brain development before, during, and after the onset of obesity, as well as family-based or genetically informed designs that can help address confounding by shared genetic and environmental factors. Accordingly, the Conclusion has been revised to clearly distinguish findings supported by longitudinal evidence from hypotheses derived primarily from cross-sectional studies.

While multimodal MRI has significantly advanced our understanding of the brain in obesity, several gaps remain that prioritize the following areas for future research: current literature is dominated by cross-sectional designs, which limits the ability to determine causality between obesity and brain alterations. Future research should prioritize large-scale longitudinal cohorts to track neurodevelopmental trajectories over time. Reliance on BMI as a crude metric often overlooks the specific impact of visceral fat or body fat distribution. Future studies should integrate multi-dimensional assessments, including visceral fat measurements and biomarkers of systemic inflammation. Given that adolescence is a critical window for both neurodevelopment and the onset of obesity, more focus is needed on how pubertal stages modulate brain-obesity associations. Moreover, Future efforts should move toward translational research, utilizing identified neural markers (e.g., prefrontal inhibition signals) to design targeted interventions. This includes exploring how weight loss, exercise, or anti-inflammatory treatments might reverse structural and functional damage to key brain regions. To improve the generalizability of findings, future studies must address the current limitations in sample size and demographic diversity across different populations.

Another important confounding factor that is often not adequately addressed in the included studies is the use of psychotropic medications. Many children and adolescents with obesity may also have comorbid psychiatric conditions, such as ADHD, depression, or anxiety, for which they receive pharmacotherapy. Psychostimulants (e.g., methylphenidate, amphetamines) have been shown to influence dopamine transmission and may alter reward-related brain activation and functional connectivity (Wu et al., 2024). Selective serotonin reuptake inhibitors (SSRIs) have been associated with changes in cortical thickness and white matter integrity. Atypical antipsychotics, which are increasingly prescribed for irritability and behavioral dysregulation, are known to induce weight gain and may have independent effects on brain structure, particularly in the prefrontal cortex and striatum. One study found that pediatric obesity is particularly common in children treated with antipsychotics, and antipsychotic exposure can increase cardiometabolic risk by increasing adiposity (Nicol et al., 2015). Thus, the observed neuroimaging differences between children with obesity and normal-weight controls may be partly attributable to medication effects rather than to obesity per se. Future studies should systematically assess and adjust for psychotropic medication use, or conduct sensitivity analyses restricted to medication-free participants, to isolate the specific effects of obesity on the brain.

## 6 Conclusion

In conclusion, this systematic review synthesized current multimodal MRI evidence on brain structural and functional changes in children and adolescents with obesity. Findings supported by longitudinal evidence include the observation that higher baseline BMI is associated with accelerated decline in prefrontal gray matter volume over time and reduced neurite density in white matter tracts, indicating that obesity may exert progressive effects on brain structure during adolescence. Findings derived primarily from cross-sectional studies, and therefore requiring confirmation in longitudinal designs, include the functional “reward-control imbalance” pattern—enhanced striatal responsivity to food cues and reduced prefrontal activation during cognitive control tasks. While this functional pattern is highly consistent across cross-sectional studies, the direction of causality remains to be established, and the possibility that pre-existing differences in reward sensitivity or executive function contribute to obesity risk cannot be excluded. The structural evidence for gray matter atrophy in prefrontal and cingulate cortices, as well as white matter integrity reductions in key fiber tracts, is also predominantly derived from cross-sectional studies. Overall, the converging evidence from multimodal MRI suggests that obesity is associated with widespread alterations in brain structure and function that may reinforce maladaptive eating behaviors. However, the cross-sectional nature of most evidence underscores the urgent need for large-scale longitudinal investigations to establish temporal sequencing and causal mechanisms.

## Data Availability

The datasets presented in this study can be found in online repositories. The names of the repository/repositories and accession number(s) can be found in the article/supplementary material.
